# Characterization of m6A methylation modifications in gastric cancer

**DOI:** 10.18632/aging.205341

**Published:** 2024-01-10

**Authors:** Wei Yin, Zhanwei Huo, Jiawei Zuo, Haixiao Wang, Bi Chen, Liqing Zhou

**Affiliations:** 1 Department of Gastrointestinal Surgery, The Affiliated Huai’an Hospital of Xuzhou Medical University and The Second People’s Hospital of Huai’an, Huai’an 223300, Jiangsu, China; 2 Department of General Surgery, Lianshui People’s Hospital Affiliated to Kangda College of Nanjing Medical University, Huai’an 223300, Jiangsu, China; 3 Department of Radiotherapy, The Affiliated Huai’an Hospital of Xuzhou Medical University and The Second People’s Hospital of Huai’an, Huai’an 223300, Jiangsu, China; 4 Department of General Surgery, The Affiliated Huai’an No. 1 People’s Hospital of Nanjing Medical University, Huai’an 223300, Jiangsu, China; 5 Department of Rehabilitation, Geriatric Hospital of Nanjing Medical University, Jiangsu Province Official Hospital, Nanjing 210000, Jiangsu, China

**Keywords:** m6A, tumor microenvironment, immunotherapy, gastric cancer

## Abstract

Widely recognized as an essential epitranscriptomic modification, RNA N6-methyladenosine (m6A) is involved in both physiological and pathological processes. Here, we want to investigate m6A modification’s potential roles in gastric cancer. Gastric cancer samples were selected from TCGA-STAD and GEO (GSE84426, GSE84433) datasets. Based on 18 regulators of m6A, m6A modification patterns were thoroughly evaluated in gastric cancer samples. Principal component analysis algorithms were used to construct the m6Ascore, using which, m6A modification features in tumor somatic mutations and immune checkpoint blockade therapy were analyzed. 34 gastric cancer samples were collected to verify the effectiveness of the m6Ascore. Here, we determined three different m6A modification patterns. m6Acluster-C modification pattern presented immune activation-associated enrichment pathways and have significant survival advantages. Then, in gastric cancer, m6Ascore could act as an independent prognostic biomarker. A significant survival benefit was exhibited in patients with high m6Ascore. Moreover, the modification signature of m6A uncovered in this study would help to predict immune checkpoint blockade therapy’s responses. In conclusion, our discoveries all pointed to the fact that modification patterns of m6A were linked to the TME. Moreover, evaluation of individual tumor’s m6A modification pattern will help to guide immunotherapy strategies that shows more therapeutic effects.

## INTRODUCTION

Gastric cancer, the fifth most common cancer in the world, ranked fourth in the most common causes of cancer-related death. In 2020, 1,089,103 new incidence of gastric cancer and 768,793 deaths related to gastric cancer were recorded (equating to one in every 13 deaths globally) [1, 2]. In male, the incidence rate is 2-fold higher than that in female. The American Cancer Society’s estimates for gastric cancer in the United States for 2023 are that about 26,500 new cases and about 11,130 deaths from this type of cancer [3]. Recently, it is found that in younger generations, especially those below 50-year-old, the incidence rates of gastric cancer have increased [4]. In all malignant tumors of the stomach, adenocarcinoma, the most common histologic subtype of gastric cancer, takes up more than 95% [5]. Helicobacter pylori infection is the major cause of gastric adenocarcinoma development. Other minor causes include dietary, lifestyle, metabolic, and genetic risk factors [6].

In eukaryotic mRNA, modification of N6-methyladenosine (m6A) is considered as a ubiquitous modification type with important biological functions [7]. m6A recognition proteins, which is able to mediate the process of splicing, degradation, exonucleation, maturation, and translation, can specifically recognize and bound m6A modified RNA. The group of m6A proteins can be further divided into: (1) methylases (‘writers’; RBM15, RBM15B, METTL 3, METTL 14, METTL 16, VIRMA, WTAP, ZC3H13) that catalyze S-adenosyl methionine groups’ transfer to RNA adenine bases; (2) demethylases (‘erasers’; ALKBH5, FTO) which are capable of reversing the methylation process; and (3) ‘readers’ (HNRNPC, FMR1, RBMX, YTHDC1, YTHDC2, HNRNPA2B1, IGFBP1, IGFBP2, IGFBP3, YTHDF1, YTHDF2, YTHDF3, LRPPRC) whose functions include m6A RNA modification recognition and downstream regulatory pathways’ activation [8–11]. Across various cancer types, m6A modification dysregulation is closely related in their drug resistance, carcinogenesis, progression and metastatic spread. For example, METTL16 enhances cholangiocarcinoma growth through PRDM15-mediated FGFR4 expression [12]. IGF2BP1 accelerates gastric cancer development and immune escape by targeting PD-L1 [13]. KIAA1429 promoted ovarian cancer aerobic glycolysis and progression through enhancing ENO1 expression [14].

In the past 10 years, immune checkpoint blockade (ICB), which includes the application of various monoclonal antibodies that inhibit PD-L1, cytotoxic T-lymphocyte antigen 4 (CTLA-4), and programmed cell death protein 1 (PD-1), has emerged for a whole spectrum of malignancies as an exciting treatment strategy [15, 16]. Unfortunately, a large portion of patients gained little and even no clinical benefit after receiving ICB, hardly meeting the clinical requirements [17, 18]. Immunotherapy’s effectiveness may be affected by various factors, which include the tumor microenvironment (TME). Apart from the cancer cells and their surrounding stroma (which comprises fibroblasts, mesenchymal cells, pericytes, and endothelial cells), TME also consists of adaptive immune cells (T and B lymphocytes) and innate immune cells (including neutrophils, macrophages, myeloid-derived suppressor cells, mast cells, natural killer cells, and dendritic cells) [19–21]. The tipping direction of balance and the question whether antitumor immunity or tumor-promoting inflammation will ensue are dictated by various immune mediators and modulators’ expression as well as the activation state and abundance of different TME cell types [22–25]. Therefore, thoroughly analyzing TME landscape’s complexity and heterogeneity can improve our capability in guiding and predicting patient’s response to immunotherapy.

Recently, the special connection between modification of m6A and infiltrating immune cells of TME has been revealed by multiple studies. For example, recruitment of PD-L1+macrophage as well as HCC cell proliferation and metastasis are promoted by ALKBH5 [26]. Expansion of γδ T cells, which enhances the anti-gastrointestinal-infection ability, is specifically induced by mA demethylase ALKBH5 depletion in lymphocytes [27]. Apoptosis of double-positive thymocytes is upregulated by loss of METTL14-dependent mA modification, following which rearrangements of Vα14-Jα18 gene are decreased, leading to a significant iNKT number reduction in the thymus and periphery [28].

In this study, collected from TCGA-STAD and GEO datasets, gastric cancer samples’ genome data were integrated to thoroughly evaluate patterns of m6A modification, which showed close correlation with TME cell-infiltrating characteristics. We also constructed a scoring system for the quantification of individual patient’s m6A modification patterns.

## METHODS

### STAD dataset source and preprocessing

In The Cancer Genome Atlas (TCGA) database and Gene Expression Omnibus (GEO), public gene-expression data and full clinical annotation were thoroughly searched. In further evaluation, we excluded patients without survival information. Then transcripts per kilobase million (TPM) values were transformed from FPKM values. We also acquired somatic mutation data from TCGA database.

### Extraction of expression levels of m6A regulators

We summarized the intersect genes of from GSE84426, GSE84433 and TCGA-STAD, and corrected these combined data using “sva” package. A total 18 m6A regulators were extracted from GSE84426, GSE84433, and TCGA data, including 2 erasers (ALKBH5 and FTO), 4 writers (METTL3, WTAP RBM15, and RBM15B) and 12 readers (HNRNPC, YTHDC1, YTHDC2, IGFBP1, IGFBP2, IGFBP3, LRPPRC, YTHDF1, YTHDF2, YTHDF3, RBMX and FMR1) (Supplementary Table 1). Then, identification of different m6A modification patterns based on 18 m6A regulators’ expression as well as patient classification was conducted using unsupervised clustering analysis for further analysis. Consensus clustering algorithm was sued to determine cluster numbers and their stability. In the above steps, the “ConsensuClusterPlus” package was used.

### Gene set variation analysis (GSVA) and functional annotation

Using “GSVA” R packages, GSVA enrichment analysis was performed for the investigation into the difference on biological process between patterns of m6A modification. From GSEA/MSigDB database, we downloaded the “c5.go.v7.5.1.symbols” gene sets and used them for GSVA analysis. Adjusted P less than 0.05 was considered as statistically significance.

### Differentially expressed genes (DEGs) identification between m6A distinct phenotypes

Based on 18 m6A regulators’ expression, patients were categorized into three distinct patterns of m6A modification using the “ConsensuClusterPlus” package for the identification of m6A-related genes. The determination of DEGs between different patterns of modification was conducted using the empirical Bayesian approach of limma R package. Criteria for the determination of DEGs were set as adjusted *P* value < 0.001.

### Generation of m6A gene signature

A set of scoring system was built to evaluate the individual gastric cancer patients’ m6A modification patterns for individual tumor’s m6A modification pattern quantification. We termed the m6A gene signature as m6Ascore. The m6A gene signature’s establishment procedures were as follows: The intersect genes were extracted from DEGs identified from different m6Aclusters (Supplementary Table 2). Thereafter, for each intersect genes within the signature, the prognostic analysis was performed under the univariate Cox regression model. For further analysis, we then extracted genes with significant prognosis (Table 1). Finally, for the construction of m6A-relevant gene signature, principal component analysis (PCA) was conducted.

**Table 1:** The prognostic analysis for intersect genes using univariate Cox regression model.

**ID**	**HR**	**HR.95L**	**HR.95H**	***p*-value**
IGFBP2	1.085739	1.016901	1.159237	0.013838
IGFBP3	1.179067	1.072126	1.296675	0.000685
CYB5B	0.839735	0.711839	0.99061	0.038277
BATF2	0.825041	0.744878	0.913832	0.000226
SUSD2	1.155556	1.042095	1.28137	0.006108

### Sample collection

34 gastric cancer tissues were obtained through biopsy or surgical procedures. The diagnosis depends on pathological results. All patients signed informed consent forms, and the study was approved by the Ethics Committee of The Affiliated Huaian No. 1 People’s Hospital of Nanjing Medical University.

### Collection of immune-checkpoint blockade information

The Cancer Immunome Atlas (TCIA; https://tcia.at/home) was used to download STAD immunophenoscore (IPS) data.

### Statistical analysis

Distance correlation and Spearman analysis were applied to compute correlations coefficients between m6A regulators’ expression the infiltrating immune cells of TME. Using One-way ANOVA and Kruskal-Wallis test, we compared the differences of three or more groups. Each dataset subgroup’s cut-off point was determined based on the correlation of m6Ascore and patients’ survival using the survminer R package. We used the “surv-cutpoint”, which is designed to repeatedly test all potential cut points to find the maximum rank statistic, to dichotomize m6Ascore, and then based on the selected maximum log-rank statistics, we divided patients into high and low m6Ascore groups to lessen the batch effect during calculation. Using Kaplan-Meier method, survival curves for the prognostic analysis were generated and identification of significant differences was conducted using the log-rank tests. All two-sided *P* value < 0.05 was accepted as statistically significance. All data were processed using R 4.1.3 software.

### Data availability

The data presented in this study are openly available in the TCGA database and GEO database.

## RESULTS

### Landscape of m6A regulators’ genetic variation in gastric cancer

**Figure 1:**
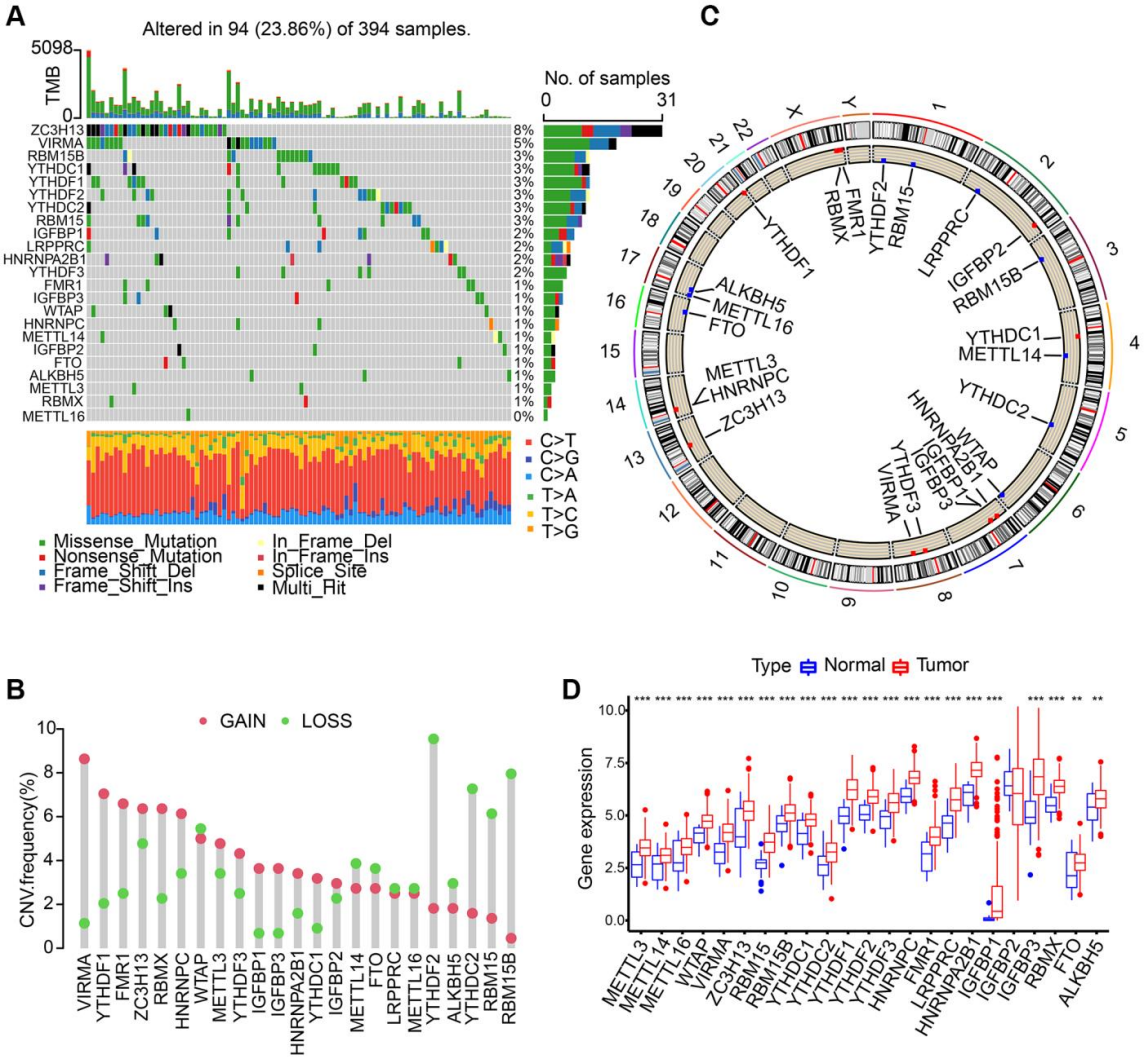
**Landscape of genetic variation of m6A regulators in gastric adenocarcinoma (STAD).** (**A**) The mutation frequency of 23 m6A regulators in 394 patients with gastric cancer from TCGA-STAD cohort. Each column represented individual patients. (**B**) The CNV variation frequency of m6A regulators in TCGA-STAD cohort. (**C**) The location of CNV alteration of m6A regulators on 23 chromosomes using TCGA-STAD cohort. (**D**) The expression of 23 m6A regulators between normal tissues and STAD tissues. The asterisks represented the statistical *p* value (^**^P < 0.01; ^***^P < 0.001).

We discovered 23 m6A regulators in total which include 8 writers, 2 erasers, and 13 readers. First, somatic mutations and the incidence of copy number variations of these 23 regulators in gastric cancer were summarized. In the collected 394 samples, there were 94 experienced m6A regulator mutations, whose frequency was calculated to be 23.86%. We discovered that the two demethylases (ALKBH5 and FTO) showed a little mutation in gastric cancer samples whilst the highest mutation frequency was exhibited by ZC3H13, followed by VIRMA (Figure 1A). In the exploration into frequency of CNV alteration, a prevalent CNV alteration in 23 regulators was discovered, most of which were copy number amplification, while METTL14, YTHDF2, FTO, ALKBH5, YTHDC2, RBM15, RBM15B, and WTAP showed dispersed CNV deletion frequency (Figure 1B). m6A regulators’ CNV alteration location on chromosomes is marked in Figure 1C. Then, based on TCGA-STAD database, we investigated the expression levels of m6A regulators’ mRNA between gastric cancer and normal samples, and discovered that compared to normal gastric tissues, 22 m6A regulators except IGFBP2 showed higher expression in tumor tissues (Figure 1D).

### 18 regulators mediating patterns of m6A methylation modification

**Figure 2:**
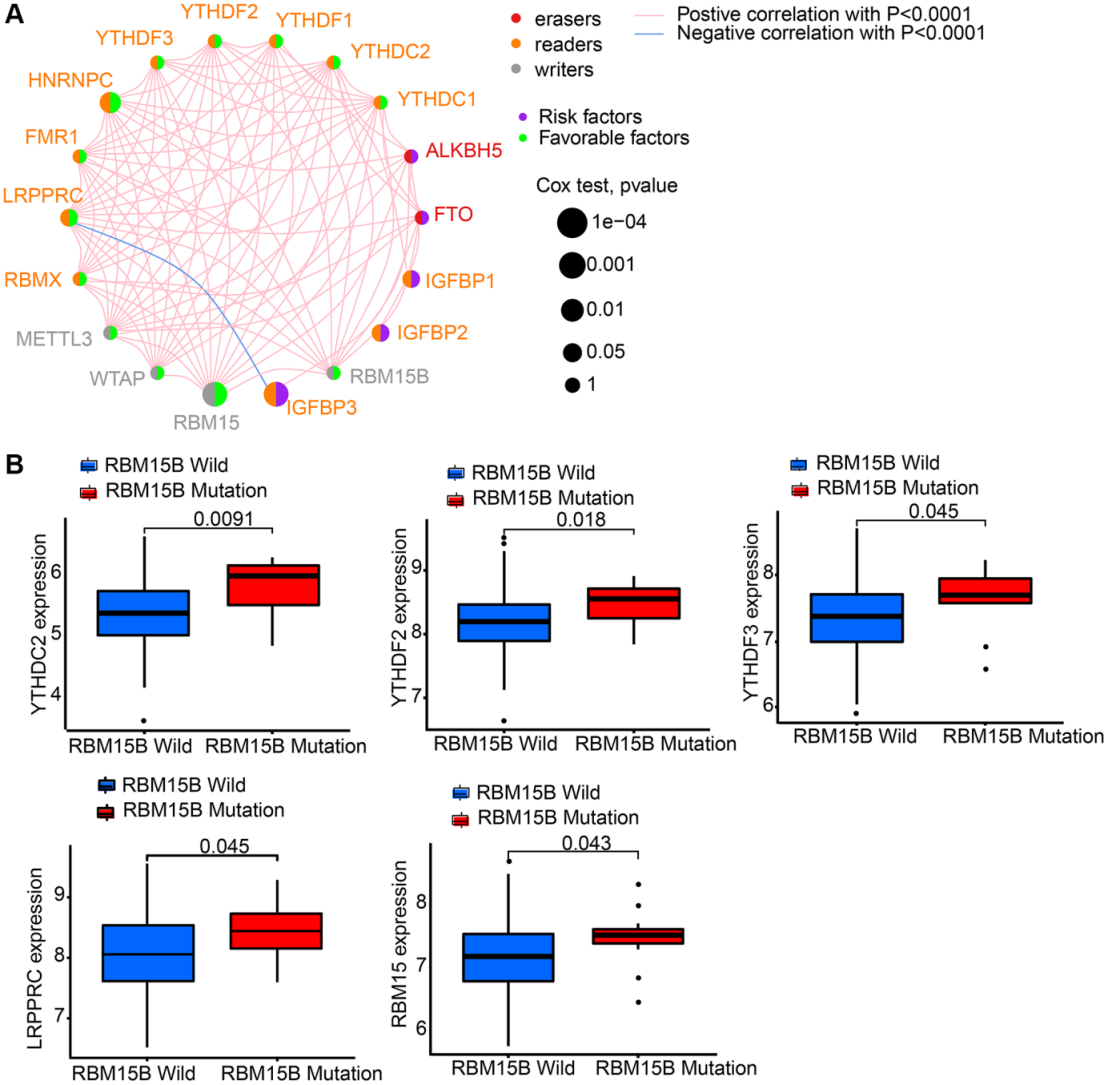
**The interaction between m6A regulators in gastric cancer.** We got intersect genes and their expression levels from GSE84426, GSE84433, and TCGA data. Then, we extracted the expression of 18 m6A regulators from the intersect genes. (**A**) The interaction between 18 m6A regulators in gastric cancer based on GSE84426, GSE84433, and TCGA-STAD cohort. (**B**) Difference in the m6A regulators expression between RBM15B-mutant and wild types.

GEO datasets, including GSE84426 and GSE84433, which consist of available clinical information and overall survival data were download. Then we got intersect genes and their expression levels from GSE84426, GSE84433, and TCGA data. Then, 18 m6A regulators’ expression levels were extracted from the intersect genes, including 2 erasers (ALKBH5 and FTO), 4 writers (RBM15, RBM15B, WTAP and METTL3), and 12 readers (HNRNPC, YTHDC1, YTHDC2, IGFBP1, IGFBP2, IGFBP3, LRPPRC, YTHDF1, YTHDF2, YTHDF3, RBMX and FMR1) (Supplementary Table 1). 18 m6A regulators’ prognostic potential were revealed by univariate Cox regression in gastric cancer patients (Supplementary Figure 1). It is shown by results that high expression of FTO, IGFBP1, IGFBP2, and IGFBP3 had worse survival rates. While, high level of HNRNPC, LRPPRC, RBM15, RBMX, and WTAP had better survival rates. Using a m6A regulator network, we depicted the comprehensive landscape consisting of m6A regulator connections, regulator interactions and their prognostic value for gastric cancer patients (Figure 2A). A remarkable correlation was found in the expression of m6A regulators belonging to the same functional group, moreover, readers, writers, and erasers showed a clear correlation. Since writer gene RBM15B demonstrated relatively higher mutation frequency, eraser gene’s expression difference between mutant and wild types of RBM15B was analyzed. Results showed that compared to wild-type tumors, in mutant tumors, LRPPRC, RBM15, YTHDC2, YTHDF2, and YTHDF3 were promoted (Figure 2B).

**Figure 3:**
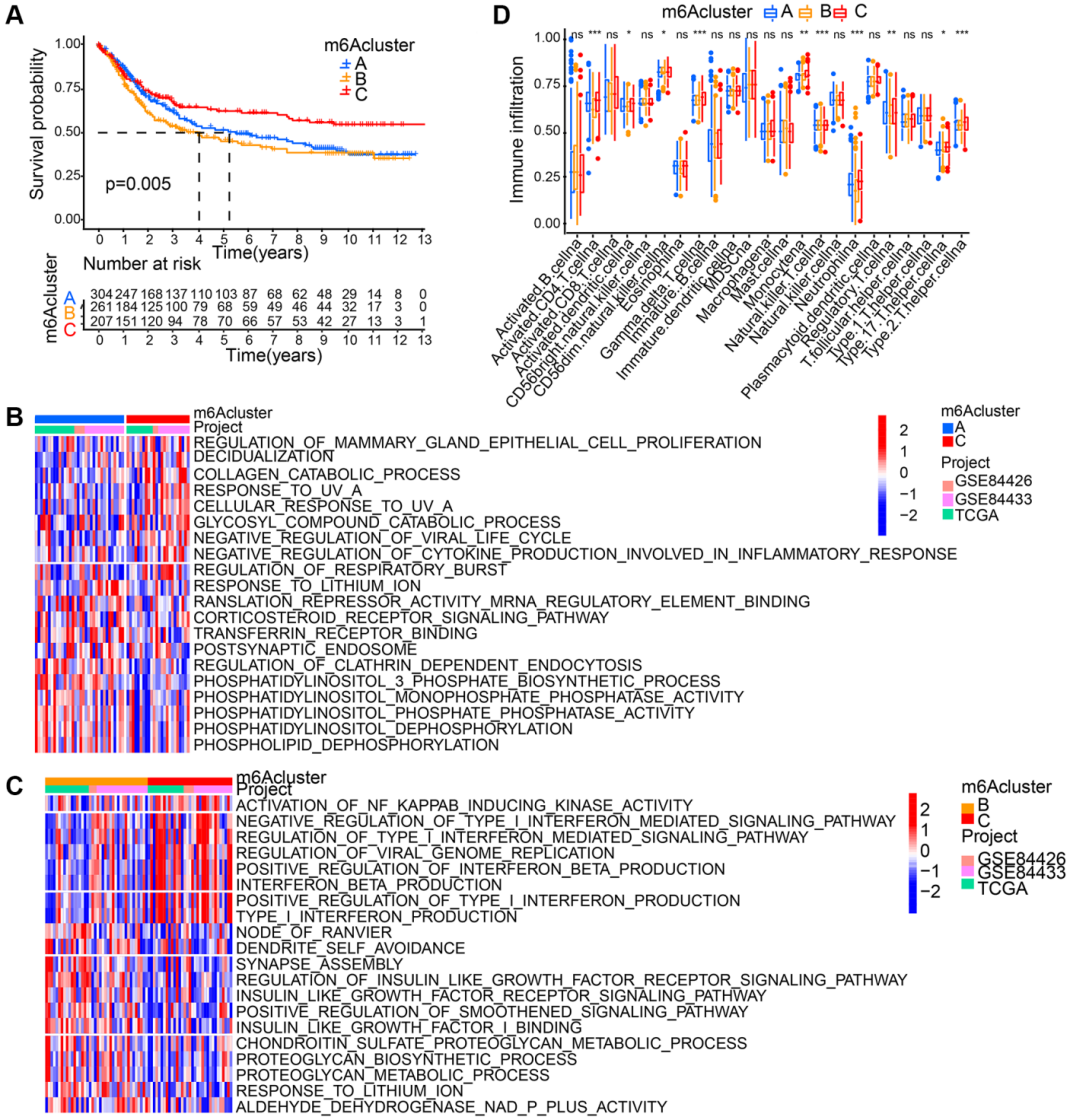
**Patterns of m6A methylation modification and biological characteristics of each pattern.** (**A**) Survival analyses for the three m6A modification patterns based on gastric cancer patients from GSE84426, GSE84433, and TCGA data. Kaplan-Meier curves with log-rank *p* value 0.005 showed a significant survival difference among three m6A modification patterns. (**B**, **C**) GSVA enrichment analysis showing the activation states of biological pathways in distinct m6A modification patterns. (**D**) The abundance of each TME infiltrating cell in three m6A modification patterns. The asterisks represented the statistical *p* value (^*^P < 0.05; ^**^P < 0.01; ^***^P < 0.001; ns: no significance).

Based on 18 m6A regulators’ expression, patients with qualitatively different patterns of m6A modification were classified using The R package of ConsensusClusterPlus, and eventually, we identified three different patterns of modification through unsupervised clustering, including pattern A (305 cases), pattern B (262 cases) and pattern C (209 cases). Respectively, these distinct patterns were named as m6Acluster A-C, (Supplementary Figure 2A). Moreover, among three different m6A modification patterns, m6A transcriptional profile depicted significant distinction (Supplementary Figure 2B). A significantly prominent survival advantage was revealed in the m6Acluster-C modification pattern by prognostic analysis conducted on the three major modification subtypes of m6A (Figure 3A).

### TME cell’s infiltration characteristics in distinct modification patterns of m6A

GSVA enrichment analysis is was performed for the investigation into the biological behaviors among these different modification patterns of m6A. m6Acluster-A showed marked enrichment in cell-cell signaling pathways including corticosteroid receptor signaling pathway, postsynaptic endosome, phosphatidylinositol 3 phosphate biosynthetic process, phosphatidylinositol dephosphorylation, phospholipid dephosphorylation, translation repressor activity mRNA regulatory element binding, and phosphatidylinositol monophosphate phosphatase activity (Figure 3B). m6Acluster-B presented enrichment pathways associated with proteoglycan metabolic pathway (regulation of proteoglycan biosynthetic process, proteoglycan metabolic process, insulin like growth factor I binding, and insulin like growth factor receptor signaling pathway). Enrichment of immune activation-associated pathways presented by m6Acluster-C included activation of NF KAPPAB inducing kinase activity, positive production regulation of interferon beta, positive type I interferon, type I interferon and interferon beta as well as negative viral life cycle regulation (Figure 3B, 3C). Analyses on TME cell infiltration suggested that m6Acluster-C showed remarkable enrichment in immune activation as well as both infiltration of adaptive and innate immune cell compared with m6Acluster-A and m6Acluster-B, including activated CD4 T cell, activated dendritic cell, Natural killer T cell, Monocyte, Gamma delta T cell, Regulatory T cell, T helper cell, and Neutrophil (Figure 3D).

### Generation of m6A gene signatures

**Figure 4:**
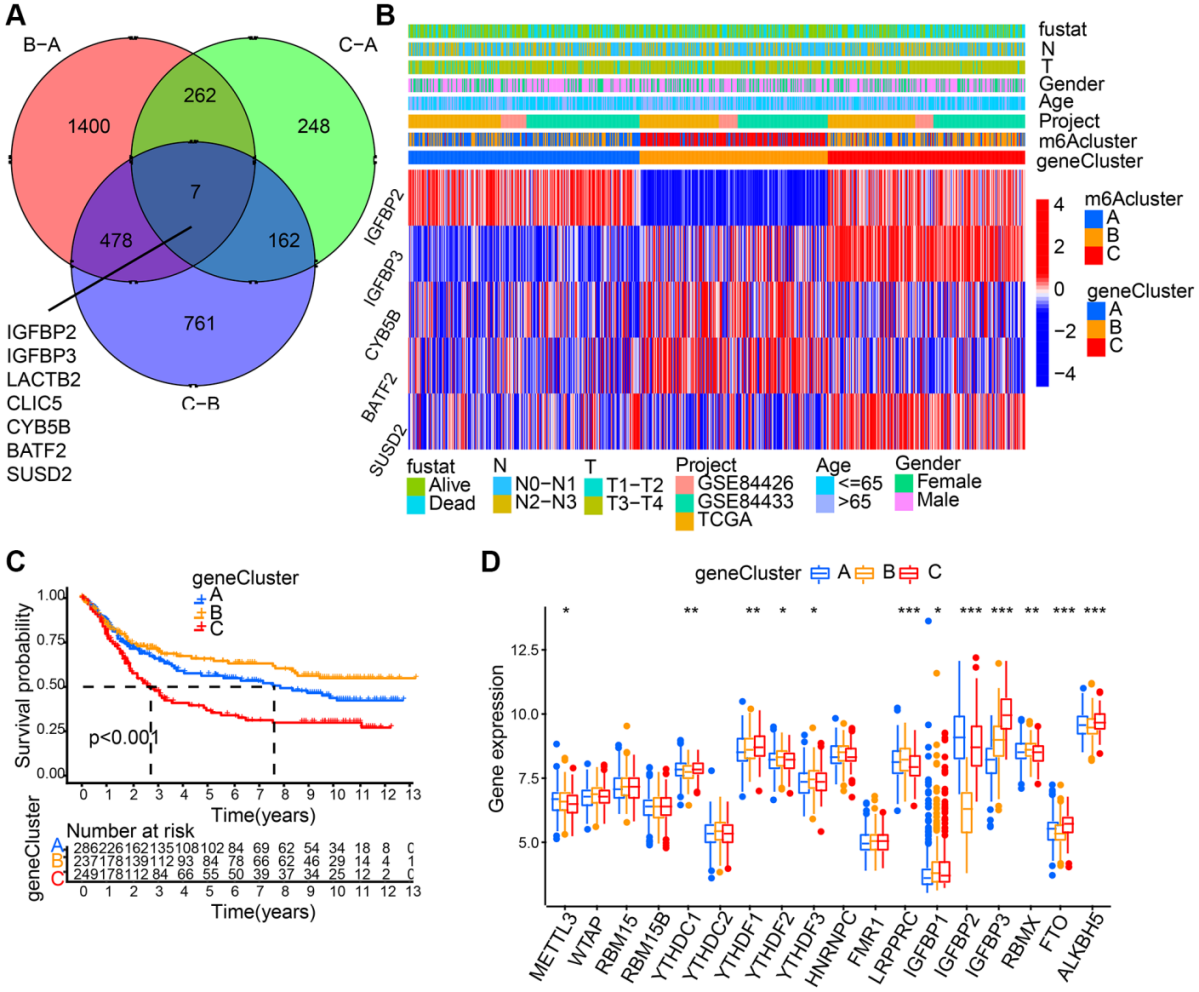
**Generation of m6A gene signatures.** (**A**) 7 m6A phenotype-associated genes shown in Venn diagram. (**B**) 5 m6A phenotype-associated genes (IGFBP2, IGFBP3, CYB5B, BATF2, and SUSD2) with the significant prognosis were used to classify patients into different genomic subtypes, termed as m6A gene cluster (**A**–**C**), respectively. The last 5 rows mean IGFBP2, IGFBP3, CYB5B, BATF2, and SUSD2, respectively. (**C**) Survival analyses for the three gene cluster. (**D**) The expression of 18 m6A regulators in three gene cluster. The asterisks represented the statistical *p* value (^*^P < 0.05; ^**^P < 0.01; ^***^P < 0.001).

7 m6A phenotype-associated intersect DEGs were determined using the limma package to further explore each m6A modification pattern’s potential biological behavior (Figure 4A). Then, prognostic analysis for each intersect gene, by means of univariate Cox regression, was performed. In Table 1, the 5 genes showing significant prognosis were listed. Then, with the 5 m6A phenotype-associated genes (IGFBP2, IGFBP3, CYB5B, BATF2, and SUSD2) as a basis, we used unsupervised clustering analysis to categorize patients into different genomic subtypes. Results from the unsupervised clustering algorithm were in accordance with m6A modification patterns’ clustering grouping, which also uncovered three different m6A modification genomic phenotypes and respectively, these three clusters were named m6A gene cluster A-C (Figure 4B). This suggests that in gastric cancer, three different patterns of m6A methylation modification indeed exist. 237 out of 776 gastric cancer patients were grouped in gene cluster B, which were proved to be associated with better prognosis, while patients from gene cluster C (249 patients) had poorer prognosis. The 286 patients from gene cluster A had intermediate prognosis (Figure 4C). Prominent differences of m6A regulators’ expression in the three m6A gene clusters was discovered, which showed consistency with the expected modification patterns of m6A methylation (Figure 4D).

### Generation of m6Ascore

**Figure 5:**
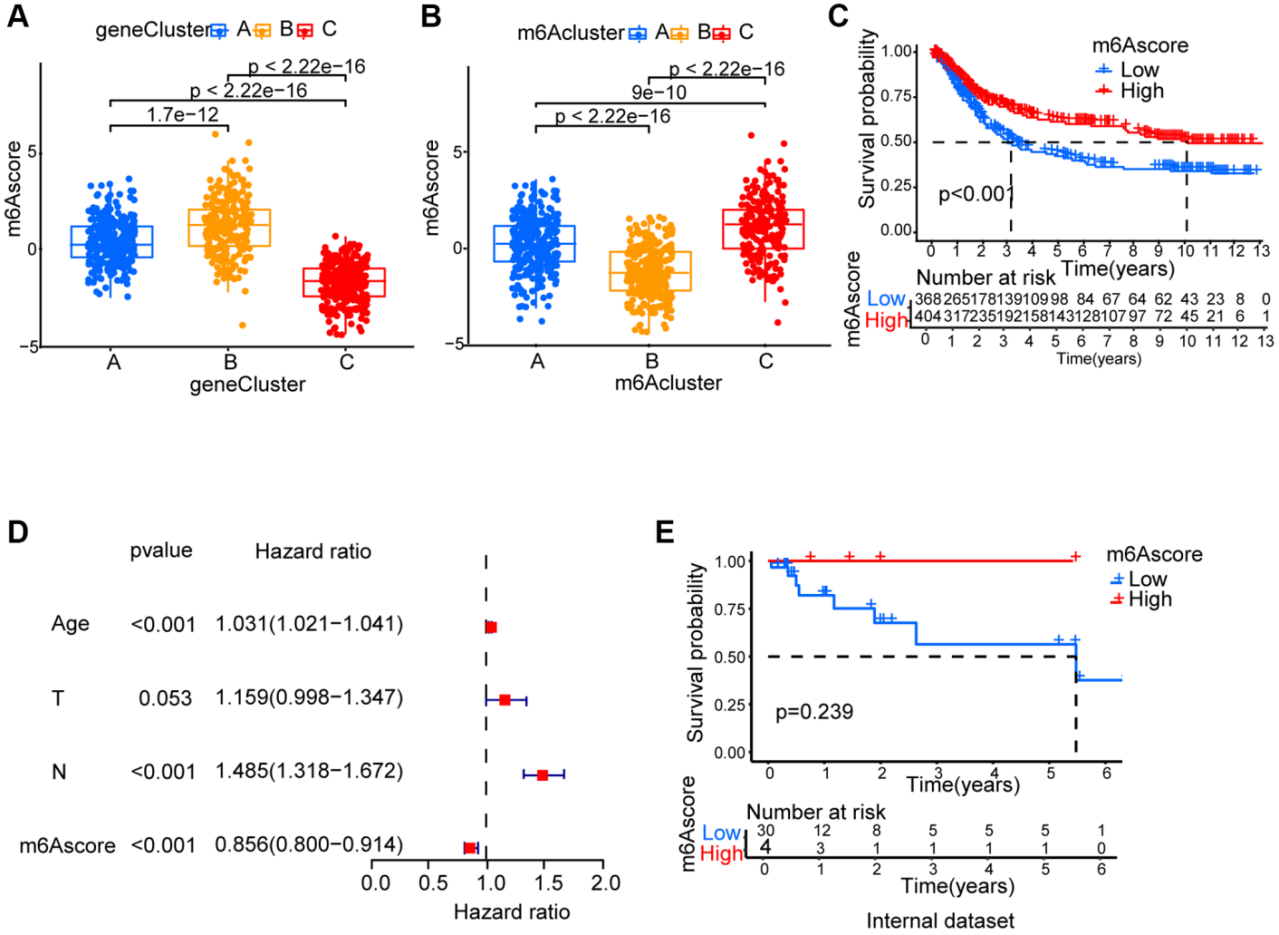
**Generation of m6Ascore.** (**A**) Differences in m6Ascore among three gene clusters. (**B**) Differences in m6Ascore among three m6A modification patterns. (**C**) Survival analyses for low and high m6Ascore patient groups. (**D**) Multivariate Cox regression analysis for m6Ascore shown by the forest plot. (**E**) We collected 34 gastric cancer patients and performed survival analyses for high and low m6Ascore groups.

Since m6A modification features individual heterogeneity and high complexity, a set of scoring system, termed m6Ascore, was built using R 4.3.1. to perform principal component analysis (PCA) based on these phenotype gene, in order to quantify the m6A modification pattern of individual gastric cancer patients. A significant difference between different m6A gene clusters on m6Ascore was found. The lowest median score was shown in gene cluster C while the highest was depicted in gene cluster B (Figure 5A). The fact that compared to the other clusters, m6Acluster C had significantly increased m6Ascore and the lowest median score was presented by m6Acluster B (Figure 5B). Next, the value of m6Ascore that can predict patients’ outcome was further identified. Using survminer package, the cutoff value of -0.101674 was determined, based on which low or high m6Ascore group was formed. Patients belonging to the high m6Ascore group showed a clear survival benefit (Figure 5C, Supplementary Table 3). Furthermore, m6Ascore’s potential as gastric cancer’s independent prognosis-predicting biomarker was tested. With factors including patients’ age, gender, T status and N status, m6Ascore was confirmed as an independent prognostic biomarker for evaluating patient outcomes by multivariate Cox regression model analysis (Figure 5D). We collected 34 STAD patients to verify the effectiveness of the m6Ascore. As showed in Figure 5E and Supplementary Table 4, patients belonging to the high m6Ascore group showed better survival rates, although the *P*-value was not statistically significant, possibly due to insufficient sample size.

### m6A modification’ characteristics in tumor somatic mutation

**Figure 6:**
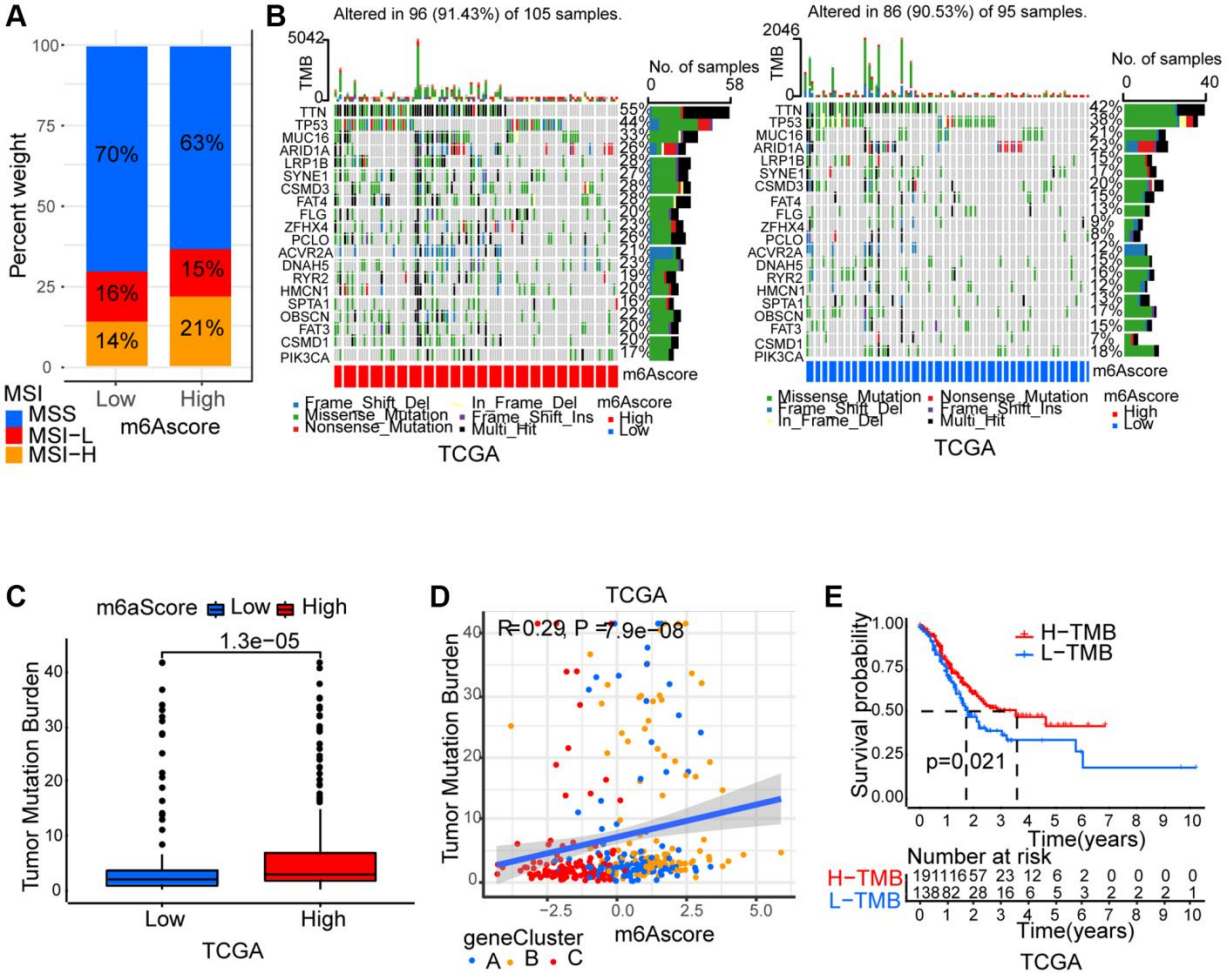
**Characteristics of m6A modification in tumor somatic mutation.** (**A**) Differences in microsatellite subtypes among high m6Ascore and low m6Ascore. MSS, microsatellite stable; MSI-H, high microsatellite instability; MSI-L, low microsatellite instability. (**B**) The waterfall plot of tumor somatic mutation established by those with high m6Ascore and low m6Ascore in TCGA-STAD cohort. Each column represented individual patients. (**C**) Differences in TMB among high m6Ascore and low m6Ascore in TCGA-STAD cohort. (**D**) The m6Ascore and TMB exhibited a significant positive correlation in TCGA-STAD cohort. (**E**) Survival analyses for high TMB and low TMB patient groups in TCGA-STAD cohort.

Thereafter, we analyzed m6Ascore and microsatellite instability (MSI)’s association. Results showed that MSI-H, which features better prognosis, took up a higher ratio in the high m6Ascore group than in the low m6Ascore group (Figure 6A). Then, using the maftools package, the somatic mutation distribution differences between high and low m6Ascore in TCGA-STAD cohort were analyzed. More extensive tumor mutation burden was exhibited by high m6Ascore group than the low m6Ascore group (Figure 6B). The fact that high m6Ascore tumors showed marked correlation with a higher TMB was confirmed by the TMB quantification analyses (Figure 6C). An evident positive correlation was discovered between m6Ascore and TMB as well (Figure 6D). Moreover, in patients with high TMB, a prominent benefit in survival was found (Figure 6E).

### Characteristics of m6A modification in the immunotherapy

**Figure 7:**
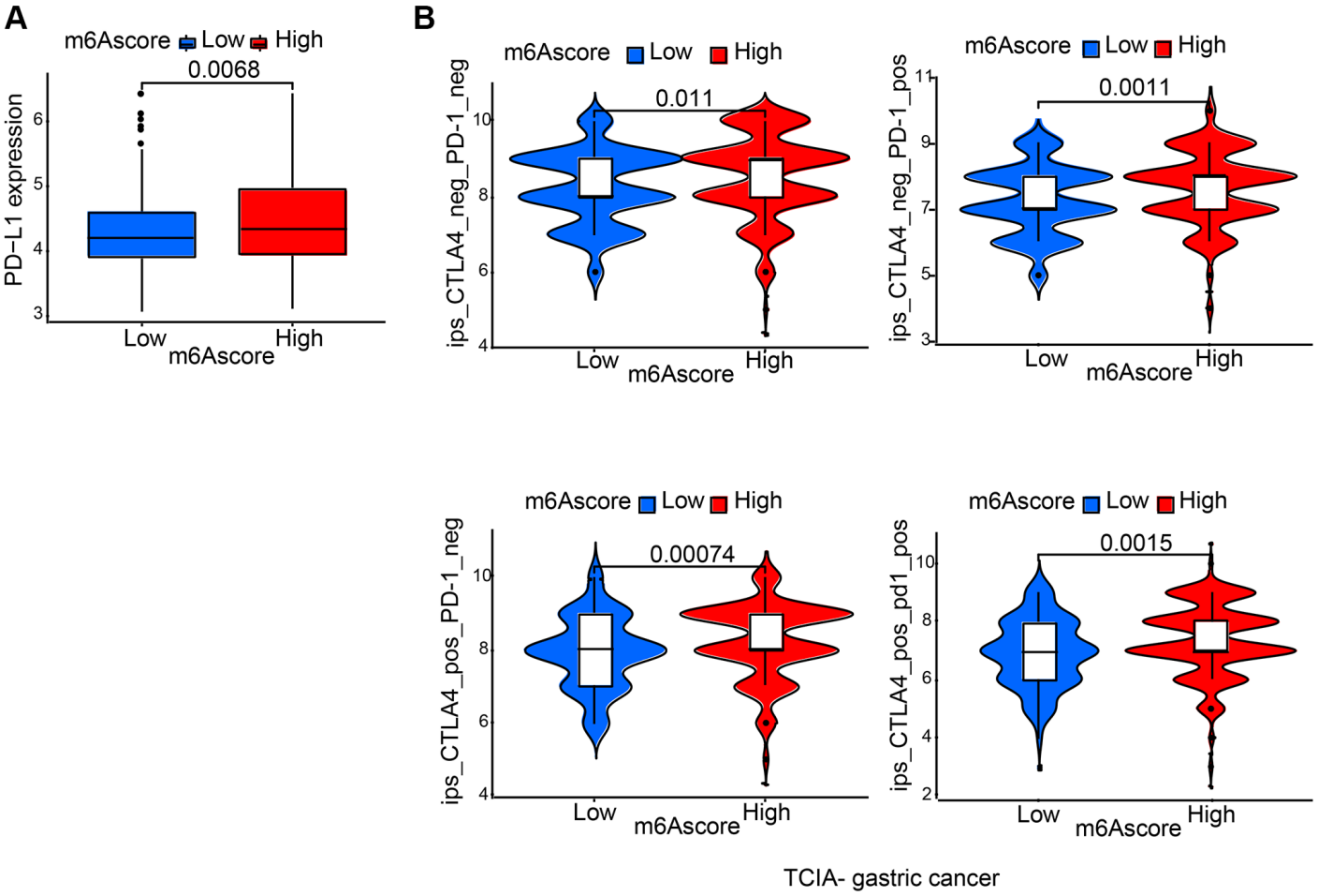
**Characteristics of m6A modification in the immunotherapy.** (**A**) Differences in PD-L1 expression between low and high m6Ascore groups. (**B**) Differences in immunophenoscore among high and low m6Ascore from TCIA (https://tcia.at/home).

It was demonstrated by further results that PD-L1 showed differential expression levels between high and low m6Ascore group, in the former of which high expression was detected (*p* = 0.0068; Figure 7A), suggesting a possible response to anti-PD-1/L1 therapy. Therefore, whether prediction of patients’ response to ICB can be done by the signature of m6A modification was investigated. From The Cancer Immunome Atlas (TCIA; https://tcia.at/home), immunophenoscore (IPS) data of gastric cancer was downloaded. Analysis demonstrated that compared with patients treated with anti-CTLA4 and/or anti-PD-1 treatment from low m6Ascore group, those from high m6Ascore group had better outcomes (Figure 7B).

## DISCUSSION

As a reversible RNA modification process, m6A RNA methylation has gained much academic focus lately. Due to the technological limits, indirect methods have been used by several studies to detect changes in m6A regulatory genes’ expression for the evaluation of relationships between human diseases and m6A status.

In this current study, 23 m6A regulators were summarized and it was discovered that compared to normal gastric tissues, 22 m6A regulators except IGFBP2 showed higher expression levels in gastric cancer tissues. Then, we got intersect genes and their expression levels from GSE84426, GSE84433, and TCGA-STAD data, from which 18 m6A regulators’ expression was extracted from the intersect genes. Based on these 18 m6A regulators, we classified patients with qualitatively different patterns of m6A modification, including m6Acluster A, B, and C. In the three major subtypes of m6A modification, a particularly prominent survival advantage in m6Acluster-C modification pattern was revealed by prognostic analysis. Enrichment pathways correlated with immune activation was presented in m6Acluster-C. Following TME cell infiltration analyses indicated innate immune cell infiltration and infiltration of adaptive immune cell and activation of immune system was remarkably enriched in m6Acluster-C than m6Acluster-A and m6Acluster-B.

Then, we extracted intersect genes from DEGs identified from different m6Aclusters. Using the univariate Cox regression model, prognostic analysis for each intersect genes in the signature was performed. We extracted the 5 genes (IGFBP2, IGFBP3, CYB5B, BATF2, and SUSD2) with the significant prognosis to constructed m6Ascore.

Referred to as readers of m6A modification, insulin-like growth factor 2 mRNA binding proteins (IGFBP2, IGFBP3) [29] are capable of recognizing and binding to m6A modification sites, thereby upregulating the translation and enhancing stability of target RNAs [30]. Reports showed that overexpression of IGFBP2 results in vessel formation [31]. IGFBP2 expression was connected to worse prognosis in glioma, colorectal cancer, lung cancer and even gastric cancer [32]. Cell migration in immortalized human endometrial stromal cells and primary human decidual stromal cells is promoted by increased expression of IGFBP3 [33]. In glioma and GBM proneural subgroup patients, higher expression level of IGFBP3 is related to shorter overall survival [34]. Epithelial-mesenchymal transition, migration, and invasion of A549 cell is enhanced by the upregulated IGFBP3 [35]. BATF2 is a novel tumor suppressor [36]. In gastric cancer, BATF2 mediated by mA modification can suppress tumor through the inhibition of ERK signaling [37]. Cancer metastasis can be promoted by SUSD2, which also induces cisplatin resistance in high grade serous ovarian cancer [38]. It is through the induction of apoptosis of Jurkat T cells that SUSD2 enhances the invasion of breast cancer cells and contributes to a possible immune evasion mechanism [39]. Shorter disease-free and overall survival are more likely to occur in hepatic recurrence of gastric cancer that shows high expression of SUSD2 [40].

It is also demonstrated by integrated analyses that in gastric cancer m6Ascore can be an independent prognostic biomarker. Our gastric cancer samples were used to verify the effectiveness of the m6Ascore. Perhaps due to insufficient sample size, the *P*-value is not statistically significant, but patients belonging to the high m6Ascore group showed significantly higher survival rates. Additionally, PD-L1 showed high expression in high m6Ascore group. IPS analysis showed that anti-CTLA4 and/or anti-PD-1 treatment had better therapeutic effects in high than low m6Ascore group. While, there still some limitations in this study: (1) the m6A modification risk scoring system should be verified by more STAD patients. (2) Whether the m6A modification risk scoring system is related to the efficacy of immunotherapy needs to be verified with clinical samples.

## CONCLUSIONS

In conclusion, an m6A modification risk scoring system, which may be applied as an independent prognostic tool or used to predict clinical outcomes of immunotherapy in gastric cancer patients, was established.

## Supplementary Materials

Supplementary Figures

Supplementary Table 1

Supplementary Table 2

Supplementary Table 3

Supplementary Table 4
