# Supplementary material for: Characterization of m6A methylation modifications in gastric cancer
Source: Aging (Albany NY). 2024 Jan 10;16(1):89–105. doi: 10.18632/aging.205341 (PMC10817395; doi:10.18632/aging.205341)
Supplement: Supplementary Figures [file aging-16-205341-s001.pdf]

SUPPLEMENTARY FIGURES

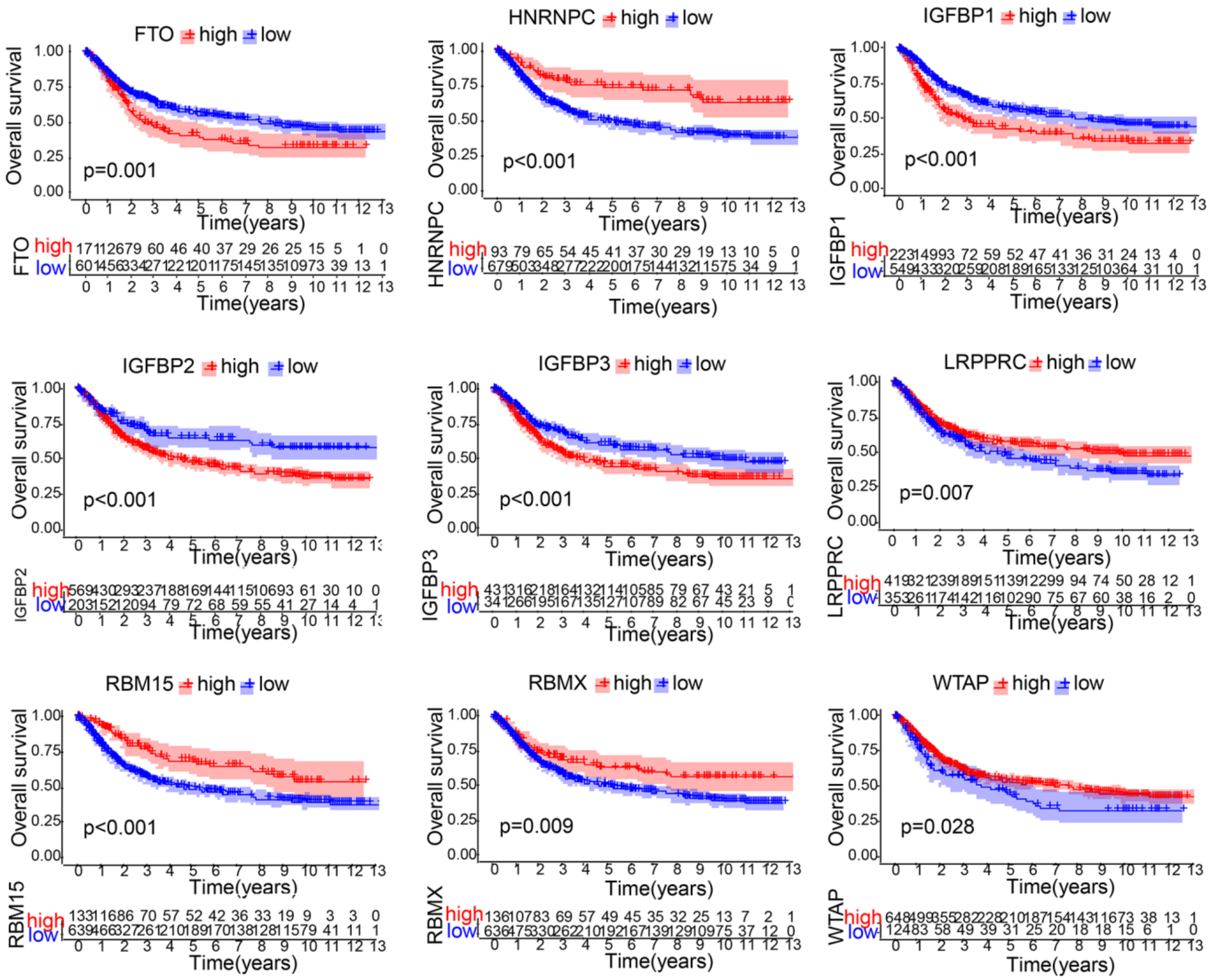

Supplementary Figure 1. 18 m6A regulators' prognostic values in gastric cancer patients.

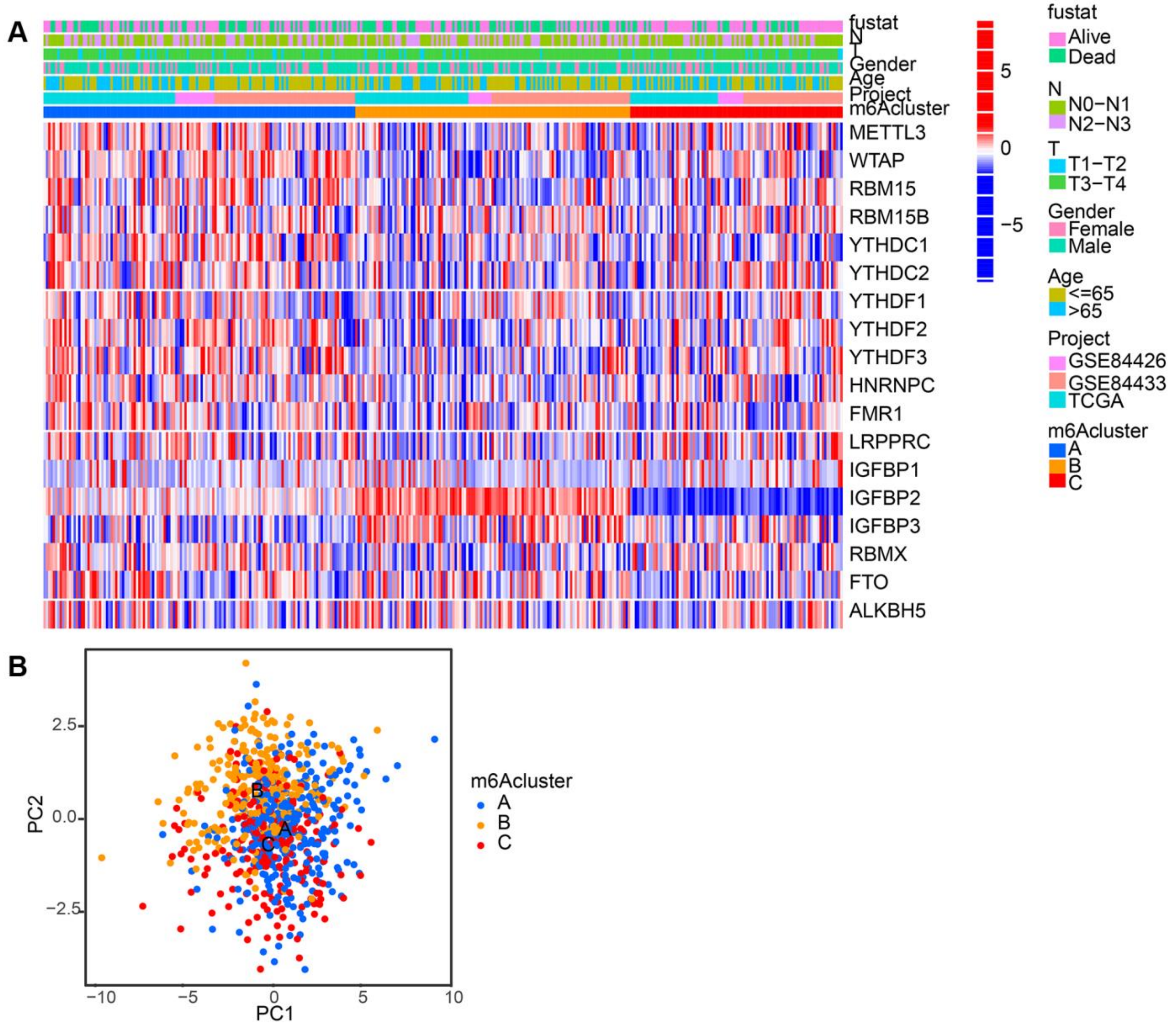

Supplementary Figure 2. Patterns of m6A methylation modification mediated by 18 regulators. (**A**) Unsupervised clustering of 18 m6A regulators in the 3 independent gastric cancer cohorts. (**B**) Principal component analysis conducted on the transcriptome profiles of three m6A modification patterns, demonstrating a significant transcriptome difference between different modification patterns.
