# Supplementary material for: Characterization of m6A methylation modifications in gastric cancer
Source: Aging (Albany NY). 2024 Jan 10;16(1):89–105. doi: 10.18632/aging.205341 (PMC10817395; doi:10.18632/aging.205341)
Supplement: Supplementary Table 4 [file aging-16-205341-s005.pdf]

SUPPLEMENTARY TABLES

Supplementary Table 4. m6Ascore of 34 gastric cancer patients.

id	futime	fustat	m6Ascore	sampleType	group
ET1	0.265753	0	–0.51758	ET1	Low
ET2	0.054795	1	–0.59115	ET2	Low
ET3	0.260274	0	0.800359	ET3	Low
ET4	0.117808	0	–1.4232	ET4	Low
ET5	0.235616	0	–1.44916	ET5	Low
ET6	0.126027	0	1.356135	ET6	Low
ET7	0.293151	0	–1.46918	ET7	Low
ET8	0.284932	0	0.026982	ET8	Low
ET9	0.410959	0	0.77102	ET9	Low
ET10	0.358904	0	–0.20958	ET10	Low
ET11	0.40274	0	–1.04344	ET11	Low
YT1	1.780822	0	–0.89751	YT1	Low
YT2	1.945205	0	6.445545	YT2	High
YT3	2.147945	0	–0.12996	YT3	Low
YT4	1.39726	0	1.739641	YT4	High
YT5	1.169863	1	–0.98456	YT5	Low
YT6	0.547945	1	–1.09723	YT6	Low
YT7	1.945205	0	–0.64187	YT7	Low
YT8	0.961644	0	0.33493	YT8	Low
YT9	0.989041	0	–1.20923	YT9	Low
YT10	0.923288	0	–0.04516	YT10	Low
YT11	0.980822	0	–0.11872	YT11	Low
YT12	1.890411	1	–0.30421	YT12	Low
YT13	2.00274	0	–0.57407	YT13	Low
YT14	0.70137	0	1.35615	YT14	High
YT15	0.353425	1	0.847103	YT15	Low
YT17	0.49589	1	–1.12134	YT17	Low
YT18	5.120548	0	–1.0457	YT18	Low
YT19	5.490411	0	–0.79212	YT19	Low
YT20	5.421918	0	5.05774	YT20	High
YT21	5.413699	0	–1.13276	YT21	Low
YT22	6.328767	0	–0.04272	YT22	Low
YT23	2.632877	1	0.043022	YT23	Low
YT24	5.476712	1	–1.9382	YT24	Low
